# Angiogenesis in Inflammatory Bowel Disease

**DOI:** 10.1155/2015/970890

**Published:** 2015-12-29

**Authors:** Canan Alkim, Huseyin Alkim, Ali Riza Koksal, Salih Boga, Ilker Sen

**Affiliations:** Department of Gastroenterology, Şişli Hamidiye Etfal Training and Research Hospital, Şişli, 34360 Istanbul, Turkey

## Abstract

Angiogenesis is an important component of pathogenesis of inflammatory bowel disease (IBD). Chronic inflammation and angiogenesis are two closely related processes. Chronic intestinal inflammation is dependent on angiogenesis and this angiogenesis is modulated by immune system in IBD. Angiogenesis is a very complex process which includes multiple cell types, growth factors, cytokines, adhesion molecules, and signal transduction. Lymphangiogenesis is a new research area in the pathogenesis of IBD. While angiogenesis supports inflammation via leukocyte migration, carrying oxygen and nutrients, on the other hand, it has a major role in wound healing. Angiogenic molecules look like perfect targets for the treatment of IBD, but they have risk for serious side effects because of their nature.

## 1. Introduction

Inflammatory bowel disease (IBD) is a chronic inflammatory disease of bowel which has two major types: ulcerative colitis (UC) and Crohn's disease (CD). They have similar and different features [1]. Although exact etiology of IBD is unknown, inappropriate immune response plays a major role in aggravation, continuation, and chronicity of inflammation [2]. Also, many genetic and environmental factors contribute to the chronic inflammation of intestinal wall [3]. Angiogenesis is an important component of both inflammation and pathogenesis of IBD [4].

Inflammation is a normal response to traumatic, pathogenic, or toxic injury in order to control and heal the damage and involves numerous cells and factors. Inflammation is tightly controlled with a balance between proinflammatory and anti-inflammatory factors [5, 6]. Normally, when a noxious agent causes a tissue injury, proinflammatory factors increase and inflammation occurs. Then, anti-inflammatory molecules increase and tissue healing occurs together with ending of inflammation. If the inflammation cannot be terminated, it passes to chronic phase. Chronic inflammation plays an important role in the pathogenesis of multiple chronic diseases, such as psoriasis, rheumatoid arthritis, osteoarthritis, metabolic syndrome, and IBD [7].

## 2. Inflammation and Angiogenesis

**Figure 1:**
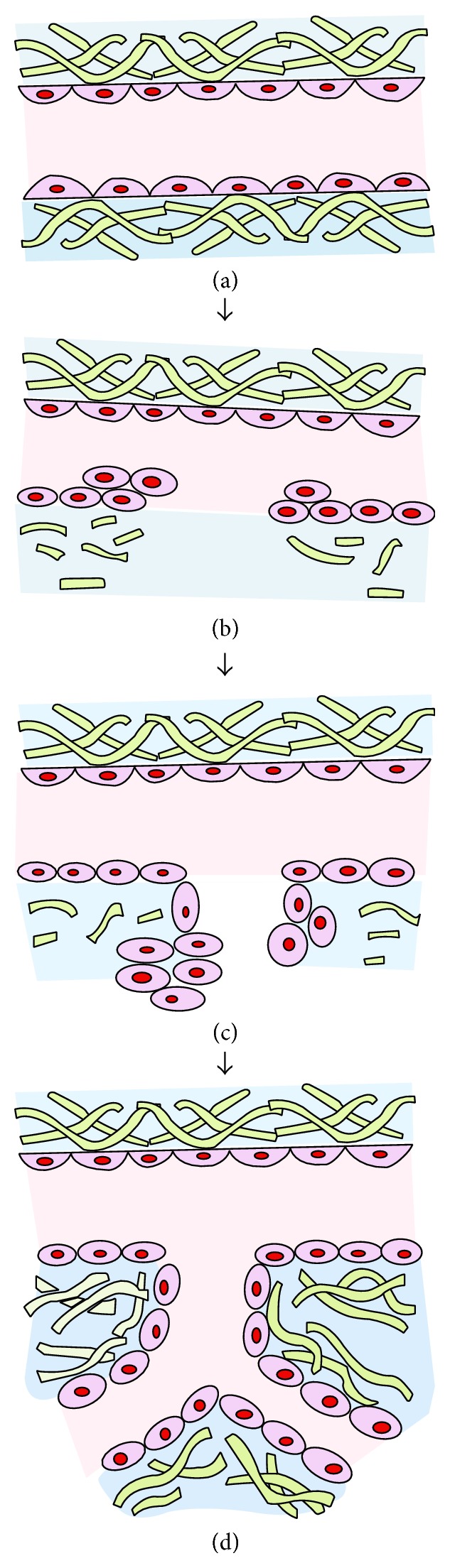
Stages of angiogenesis process. (a) Normal, quiescent intestinal vessel: endothelial cells (ECs) with smooth basal membrane and regular extracellular matrix (ECM) are seen. (b) After angiogenic stimulation, activated EC secretes various proangiogenic and proinflammatory molecules. Then, increased permeability, vasodilatation, and extravasation of leucocytes occur. Basal membrane and ECM are degraded by metalloproteinases and proteases. ECs proliferate and migrate from this degraded area. After sprouting, ECs adhere to the matrix. (c) Tubulogenesis, lumen formation, and beginning of stabilization. (d) Remodeling and maturation: angiogenesis is completed in this stage by migration of pericytes and vascular smooth muscle cells to this area. If maturation of this new vessel is not completed, angiogenesis continues as pathogenic angiogenesis.

Angiogenesis is the process of developing new vessels from initial vessels and plays an important role in wound healing, chronic inflammation, and tumor occurrence, growth, and metastasis. The angiogenesis event has multiple steps (Figure 1). These steps are stimulation of endothelial cell (EC), degradation of basal membrane and extracellular matrix (ECM), proliferation of EC, migration and adhesion of EC, tubulogenesis, stabilization, and lumen formation, remodeling, and maturation. Newly formed complex vascular network develops at the end of these steps [5, 8].

**Figure 2:**
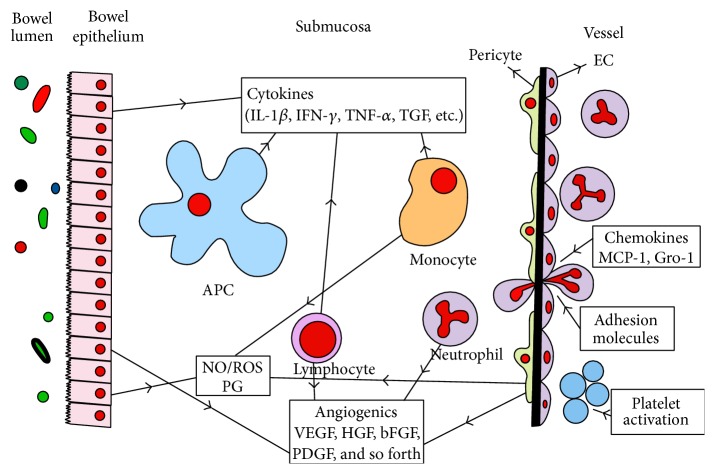
Relationship between bowel lumen, endothelium, immune cells, and vessel in immune-driven angiogenesis in inflammatory bowel disease. bFGF: basic fibroblast growth factor; Gro-1: growth-regulated oncogene-1; HGF: hepatocyte growth factor; IFN: interferon; IL: interleukin; MCP-1: monocyte chemoattractant protein; NO: nitric oxide; PDGF: platelet-derived growth factor; PG: prostaglandin; ROS: reactive oxygen species; TGF: transforming growth factor; TNF: tumor necrosis factor; VEGF: vascular endothelial growth factor.

Chronic inflammation and angiogenesis are two closely related processes. Hypoxia stimulates both of them. Macrophages and other immune/inflammatory cells migrate to the hypoxic region and secrete multiple angiogenic factors (growth factors, cytokines, proteases, and nitric oxide). These factors stimulate EC, the beginning step of angiogenesis. In turn, angiogenesis enhances inflammation by carrying oxygen, nutrients, cytokines, and adhesion molecules to the region (Figure 2). In conclusion, we can say that inflammation and angiogenesis enhance each other like a vicious circle [5, 8–10].

## 3. IBD and Angiogenesis

In the bowel of IBD patients, there is continuous ulceration (injury) and regeneration. Angiogenesis is necessary for carrying of nutrients and oxygen to the injured region, cleaning of waste products from tissue, and chemotaxis of cells. In IBD, physiological angiogenesis turns to pathological angiogenesis at early stages of the disease. Normally, neovasculogenesis is controlled by the fluctuant balance between proangiogenic and antiangiogenic molecules. After regeneration, angiogenic molecules are converted to normal status in physiological angiogenesis. But, in pathological angiogenesis, angiogenic balance does not turn to normal. The factor or factors causing conversion of physiological angiogenesis to pathological angiogenesis are still unknown. In IBD, angiogenesis is driven by inflammation and immune response; for this reason, it is called “immune-driven angiogenesis” [8, 9, 11].

The importance of vascular involvement in IBD has been known for more than four decades [12, 13]. Intestinal microcirculation has multiple crucial roles in the pathogenesis of IBD, especially in angiogenesis [14–18]. Alkim et al. [19] demonstrated the presence of enhanced microvessel density in intestinal tissue of both UC and CD patients immunohistochemically, which correlated with the disease activity and expression of VEGF. Danese et al. [20] showed that microvessel density and angiogenesis were increased in intestines of patients having IBD by narrow band imaging endoscopy method. In IBD bowel, angiogenesis can be demonstrated by dynamic contrast-enhanced ultrasonography and magnetic resonance imaging, also [21, 22]. In another study, Danese et al. [23] found increased microvessel density and *α*V*β*_3_ positive angiogenic vessels in IBD mucosa, but not in normal mucosa. Also they demonstrated that IBD mucosal extracts induce higher human intestinal microvascular endothelial cell (HIMEC) migration than normal mucosal extracts and this induction mostly depends on IL-8 (interleukin), not VEGF or bFGF (basic fibroblast growth factor). Angiogenesis is an important part of the pathogenesis of IBD. Future treatment options will be targeted to this pathway.

## 4. Angiogenic Mediators

Angiogenesis is coordinated by proangiogenic molecules and its ligands: although there are multiple factors in angiogenesis cascade, VEGF is the major angiogenic molecule [5, 8].

Family of* VEGF* has seven members as VEGF-A, VEGF-B, VEGF-C, VEGF-D, VEGF-E, VEGF-F, and placental growth factor (PlGF). They bind to three specific receptors (VEGFR1, VEGFR2, and VEGFR3). VEGF-A is the best recognized and major factor in pathological angiogenesis. VEGF-A increases vascular permeability and induces EC proliferation, directed migration, and differentiation. Also, VEGF increases adhesion of leukocytes to the endothelium and chemotaxis of monocytes. VEGF activates nuclear factor *κ*B (NF*κ*B) and causes production of many proinflammatory cytokines and chemokines. VEGF-A has got four major isoforms; VEGF_165_ is the major contributor of pathological angiogenesis and exerts its effect by binding to VEGFR2 and neuropilin-1 [24, 25].

After Schurer-Maly et al.'s [26] study, VEGF was investigated extensively in IBD. The level of VEGF was found to be increased in serum of IBD patients [26–28]. Griga et al. demonstrated that the sources of increased serum VEGF were inflamed intestinal tissue [29] and peripheral blood mononuclear cells [30] of IBD patients. Also they found that VEGF expression was markedly increased in the inflamed mucosa of both CD and UC patients, when compared with the normal mucosa of the same patient [31]. We showed that VEGF expression was increased in colonic epithelium and was higher in all IBD groups (both active and inactive CD and UC) when compared with healthy control [19, 32]. Scaldaferri et al. [33] reported that VEGF-A and VEGFR2 levels were increased in intestinal mucosal samples of IBD patients and mice with experimental colitis. In this study, they detected that VEGF-A overexpression increased the angiogenesis of HIMEC cells and endothelial adhesion of neutrophils, in vitro. Also, they found that increased VEGF-A expression increased mucosal angiogenesis and stimulated leukocyte adhesion, in vivo. Studies confirmed that VEGF-A was the major actor of the angiogenesis; only Kapsoritakis et al. [34] did not find elevated serum or plasma level of VEGF in IBD. Ferrante et al. [35] investigated VEGF polymorphisms in IBD and they found that VEGF polymorphism has no effect on susceptibility to IBD and on serum VEGF levels of IBD patients. They conclude that although increased VEGF and angiogenesis are important features of IBD, they are not determined genetically.

*Lymphangiogenesis* is a new research area in the pathogenesis of IBD. Lymphatic vessels drain proteins (cytokines, growth factors, bacterial antigens, etc.) and activated immune cells. VEGF-C and VEGF-D are involved in lymphangiogenesis, especially VEGF-C, and its receptor VEGFR3 is very important. The molecules which play a role in angiogenesis have effect in lymphangiogenesis, also [36, 37]. The presence of increased lymphatic vessels was demonstrated [38]. D'Alessio et al. [37] showed that VEGF-C provides marked protection against the development of acute and chronic colitis in animal models via increasing inflammatory cell mobilization and bacterial antigen clearance from inflamed colon. In fact, newly formed lymph vessels may reduce inflammation, but defective lymphangiogenesis cannot. It is still discussed whether lymphangiogenesis was protective or pathological in IBD [39].

*Angiopoietins* play important roles in the late phase of angiogenesis. We know four types of angiopoietins: Ang1, Ang2, Ang3, and Ang4. Their specific tyrosine kinase receptor is Tie2. When Ang1 binds to Tie2, it favors maturation of pericytes and EC and silences microvasculature. Ang2 that binds to Tie2 is inducible and secreted by the endothelium. It probably causes destabilization of blood vessels and sprouting of new vessels. It was reported that Ang2 and Tie2 were high; in contrast, Ang1 was found to be low in CD patients [39, 40]. The balance between Ang1 and Ang2 is suggested as very important in controlling of inflammation, angiogenesis, and maybe lymphangiogenesis. The relationship between Ang-Tie-2 signaling pathway and NF*κ*B may be the determinative point of the vicious circle of inflammation and angiogenesis [40–45].

*Growth factors* are very important in angiogenesis.* Platelet-derived growth factor* (PDGF) is a potent angiogenic growth factor. PDGF is secreted in response to hypoxia, thrombin, and other cytokines and growth factors. It modulates recruitment of pericytes and vascular muscle cells to the angiogenic area [46, 47]. PDGF-A and PDGF-B and their receptors alphaR and betaR were found to be increased in both active inflammation and active fibrosis areas of IBD [48].* Transforming growth factor-β* (TGF-*β*) is a multifunctional growth factor that regulates proliferation, migration, survival, and differentiation of ECs, ECM synthesis, and vascular homeostasis. It has opposite effect in angiogenesis by binding two different receptors (serine/threonine kinase receptors type I and type II). TGF-*β* was found to be increased in IBD [49, 50].* bFGF* is a heparin-binding protein, which promotes angiogenesis through EC proliferation, migration, and differentiation and mesenchymal cell proliferation. There was a significant positive correlation among the elevated serum levels of VEGF and bFGF. Overexpression of VEGF and bFGF in endothelial cells was revealed and TGF-beta was found in inflammatory cells of active IBD patients [50, 51]. It seems that bFGF and TGF-*β* contribute to fibrostenotic Crohn's disease. Syndecan-1 is activator and modulator of bFGF. It was shown that increased syndecan-1, TNF-*α*, and bFGF levels of patients with active IBD were declined after infliximab therapy [52]. In contrast, Paunovic et al. [53] demonstrated that bFGF caused mucosal healing of ulcerative colitis in rat.* Hepatocyte growth factor* (HGF) is secreted from stromal cells present within the inflammatory tissue and activated by hepatocyte growth factor activator (HGFA). HGFA is activated by thrombin in damaged tissue. HGF leads to proliferation, activation, and differentiation of EC [9, 54]. Srivastava et al. [55] found increased serum level of HGF in young IBD patients; on the other hand, Sturm et al. [56] reported that plasma HGF and TGF-*β* levels of IBD patients and healthy controls were not different. Treatment with HGF cDNA induced tyrosine phosphorylation of intestinal c-Met/HGF receptors, inhibited apoptosis, and promoted mitosis in intestinal epithelial cells, accelerating intestinal epithelial restoration and suppressing inflammation [57].

Cellular* adhesion molecules* and their ligands play important roles in the recruitment of immune cells and pathologic angiogenesis. Intercellular adhesion molecule-1 (ICAM-1, CD54)/*β*_2_-integrins, vascular cell adhesion molecule-1 (VCAM-1)/*α*_4_ *β*_1_ and *β*_2_-integrins, platelet endothelial cell adhesion molecule-1 (PECAM-1, CD31)/*α*V*β*_3_-integrin, mucosal addressing cellular adhesion molecule-1 (MadCAM-1)/*α*_4_ *β*_7_, CD146, P-selectin, E-selectin, and VE-cadherin are expressed on vascular endothelium and upregulated in angiogenesis. They coordinate the recruitment of immune cells and interaction of immune cells with the endothelial cell during angiogenesis [58, 59]. CD11b, CD18, and ICAM-2 were important for transepithelial migration of neutrophils in UC, whereas CD11a, CD11c, ICAM-1, and ICAM-3 seem central in leukocyte locomotion and aggregation in CD. Alkim et al. [60] demonstrated increased *α* chain of *β*_2_-integrins (CD11b and CD11c) in peripheral blood phagocytes of CD patients by flow-cytometry method.

Remodeling of ECM is a crucial pathogenetic mechanism for IBD. ECM is a substantial element for tissue, not only for support, which also contributes to tissue function and homeostasis. ECM is composed of fibrous proteins and glycosaminoglycans. ECM remodeling is organized by matrix proteinases (e.g., matrix metalloproteinases (MMPs) and cathepsins), lysyl oxidases (LOX/LOXL), and heparanases [61]. MMPs are extensively investigated in IBD. They are produced by ECs. MMPs are a large family ranging from MMP-1, MMP-2, and MMP-3 to MMP-9. Also they have inhibitors called tissue inhibitors metalloproteinases (TIMP): TIMP-1 and TIMP-2. MMP-2 (gelatinase A) is a homeostasis molecule and MMP-9 (gelatinase B) is proangiogenic. Lakatos et al. [62] demonstrated that serum antigen concentrations of MMP-9, TIMP-1, and TIMP-2 were significantly increased in patients with UC and CD compared to controls. They may increase angiogenesis by inhibiting vascular maturation during vessel growth [9, 62]. MMP-9 is studied widely as a fecal biomarker. In a last study, fecal MMP-9 had high sensitivity in the detection of endoscopically active UC and pouchitis. But fecal MMP-9 did not correlate with any of the activity indices of CD [63].

The* hypoxia-inducible factor* (HIF) is a heterodimeric transcription factor that forms in response to hypoxia. It has constitutive HIF-1*β* and oxygen regulated HIF-*α* subunits (HIF-1*α*, HIF-2*α*, and HIF-3*α*). The regulation of HIF-*α* by oxygen is mediated with prolyl hydroxylase. HIF-*α* is degraded by hydroxylase at normal oxygenic environment. HIFs and NF*κ*B are formed in response to hypoxia and inflammation and they may have dual effect, either protective or inflammatory. HIFs promote transcription of several angiogenic genes including VEGF and Ang2 [5, 64, 65].

The relation between immune and nonimmune cells is very important for continuation of inflammation-induced angiogenesis. Firstly, ECs can mediate immune cells and their function, thus managing innate and acquired immune systems [8, 16].* CD40-CD40 ligand* (L) pathway is important in these interactions. CD40, a transmembrane-glycoprotein member of the TNF receptor gene family, is expressed by antigen-presenting cells, monocytes, activated CD4+ T cells, platelets, and ECs. CD40-CD40L pathway interacts with angiogenesis by different ways. They stimulate EC migration and secretion of proangiogenic cytokines; they especially induce VEGF expression and VEGF induced angiogenesis [66, 67]. Both CD40- and CD40L-deficient mice were protected from DSS-induced colitis and displayed a significant impairment of gut inflammation-driven angiogenesis, as assessed by microvascular density [66]. Monocyte chemoattractant protein 1 (MCP-1) and growth-regulated oncogene-1 (Gro-1) are proinflammatory chemokines released from leukocytes. These chemokines activate EC. Sensitivity of EC to TNF-*α* and VEGF is modulated by Ang2 [8, 59].

*Nitric oxide* (NO) derived from endothelium is a relaxing factor, and also it induces VEGF-A. There are three forms of NO synthase (NOS): constitutive endothelial NOS (eNOS), inducible NOS (iNOS), and neuronal NOS (nNOS). NO produced by eNOS is anti-inflammatory and antioxidant. On the other hand, iNOS is an inducible generator of NO during disease. NO and NOS seem to have dual pathogenetic function in IBD [68]. iNOS and eNOS immunoreactive cells were significantly numerous in inflamed mucosa of UC compared to CD in Palatka et al.'s study [69]. Alkim et al. [32] found high expression of iNOS in UC and CD patients and it was correlated with VEGF expression. In Vallance et al.'s study [70], both eNOS and iNOS knockout mice developed more severe colitis compared with wild-type mice. During colitis, iNOS expression was dramatically increased on both epithelial and lamina propria mononuclear cells. Dhillon et al. [71] demonstrated the importance of NOS2 gene, which encodes iNOS, in susceptibility for younger IBD presentation.

*Caveolae* are specialized lipid rafts and act as site of regulation or as a signaling platform for several important angiogenic molecules including VEGF and eNOS and multiple proangiogenic cytokines. Caveolins (Cav-1, Cav-2, and Cav-3) are structural protein of caveolae on the plasma membrane of several cell types. Cav-1 is the major protein of caveolae in EC. In the study of Chidlow Jr. et al. [72], Cav-1 protein levels increased after the induction of colitis in wild-type mice. In Cav-1(−/−) mice or mice given Cav-1 inhibitory peptide, the colitis histopathology scores, vascular densities, and levels of inflammatory infiltrates decreased significantly compared with controls. Cav-2 was found to be increased in the inflamed mucosa of patients with UC, but not in CD or ischemic colitis [73]. Cav-1 is an inhibitor of eNOS and decreases endothelial NO production. On the other hand, production of endothelial NO depends on Cav-1, because of colocalization of eNOS and Cav-1 in caveolae. Cav-1 has potential protective role in intestinal inflammation. Cav-1 seems important for VEGF and TNF signaling in angiogenesis [9, 72, 74, 75].

*Endoglin*, a TGF-*β* superfamily coreceptor, is expressed on ECs and some myeloid cells. It is implicated in the maintenance of vascular integrity and regulation of vascular tone. Endoglin modulates angiogenesis in wound healing and probably in resolution of inflammation. Jerkic et al. [11] studied acute colitis model in Eng(+/−) and control mice, which peaked at day 9. While control mice recovered by days 19–26, Eng(+/−) mice progressed to chronic colitis and showed numerous vascular protrusions penetrating into the serosa of the inflamed distal colon. Higher VEGF levels and increased vascular permeability in the distal colon may predispose Eng(+/−) mice to progress to chronic and persistent bowel inflammation, associated with pathological angiogenesis. It was shown that anti-VEGF therapy reduced inflammation, in this experimental colitis model [76].

## 5. Natural Antiangiogenic Molecules

*Thrombospondins* (TSPs) are well-known antiangiogenic molecules. TSPs include five calcium binding extracellular glycoproteins. TSP-1 is the major antiangiogenic molecule. They decrease angiogenesis via stimulating EC apoptosis and regulate chemotaxis and inflammation [77–79]. We found higher TSP-1 and VEGF expression in IBD [32], but Wejman et al. [80] did not find TSP expression in IBD. In the study of Ortiz-Masià et al. [81], CD38 (a class B scavenger receptor) and its ligand TSP-1 were found to be upregulated by HIF-1, and this increases the phagocytosis of neutrophils by macrophages during hypoxia. Zak et al. [78] showed increased angiogenesis via higher VEGF, bFGF, and microvessel density in TSP-1(−/−) mice.

*Angiostatin* and* endostatin* are 20 and 50 kDa fragments cleaved from plasminogen and collagen XVIII, respectively. Their proteinases are MMP-2 and MMP-9. They inhibit proliferation and migration of ECs and induce apoptosis of ECs. Endostatin increases in patients with IBD and in experimental UC for balancing increased VEGF or angiogenesis [42, 82]. Deng et al. [83] reported that mesalamine treatment reverses the imbalance between the angiogenic factor VEGF and antiangiogenic factors endostatin and angiostatin in experimental UC.

*Corticotropin-releasing hormone* (CRH) family includes CRH, urocortin I, urocortin II, urocortin III, and CRH receptors (CRHR1 and CRHR2). This family modulates stress-related responses through the hypothalamic-pituitary-adrenal axis. CRHR1 promotes intestinal inflammation and angiogenesis; in contrast, CRHR2 is antiangiogenic and inhibits inflammation in experimental colitis [84, 85]. The inflamed intestines of CRHR1(−/−) mice had reduced microvascular density and reduced expression of vascular endothelial growth factor- (VEGF-) A, whereas the intestines of CRHR2(−/−) mice had increased angiogenesis and VEGF-A levels. An antagonist of VEGFR2 activity alleviated colitis in CRHR2(−/−) mice [84].

## 6. Antiangiogenic Therapies in IBD

After clarifying angiogenesis as an essential part of IBD pathogenesis, the discussion about the therapies against angiogenesis was started. As mentioned above, angiogenesis is a complex process and includes multiple cells, molecules, and pathways. All of these molecules may be a new target of therapeutic research.

While standard IBD therapy recovers disease, it has some correcting effects on pathologic angiogenesis. Mesalamine establishes angiogenic balance by reducing angiostatin and endostatin and induced TNF-*α* and MMP-9 activity in experimental colitis [83]. Rutella et al. [86] detected inhibition of angiogenesis in CD patients by infliximab therapy. Thalidomide is one of the old anti-TNF drugs. Nowadays, its effectiveness is demonstrated in refractory pediatric IBD patients. It has anti-inflammatory and antiangiogenic effects [87].

*Bevacizumab* is a monoclonal antibody against VEGF. It is used in combination therapy of colorectal carcinoma. Because of serious side effects such as inhibiting wound healing, intestinal perforation, and surgical anastomosis leakage, bevacizumab did not enter routine clinical application in IBD [88].* Sunitinib* and* sorafenib* are tyrosine kinase inhibitors that inhibit VEGFR1, VEGFR2, and VEGFR3. These drugs are used in the treatment of renal cell cancer and hepatocellular carcinoma, but they cause activation of the bowel disease in patients with IBD [89]. Boers-Sonderen et al. [90] described a patient with metastatic renal cell carcinoma and a history of Crohn's disease who was treated with sunitinib and developed severe exacerbation of Crohn's disease. After rechallenge with sunitinib, second exacerbation occurred. Antiangiogenic receptor tyrosine kinase inhibitors suppress the VEGF/VEGFR pathway but the expected decrease in colonic microvessel density did not follow, suggesting possible involvement of other angiogenic pathways [91]. In the DSS model of colitis,* DC101* is an anti-VEGFR2 monoclonal antibody and was used in experimental colitis [92]. The activation of VEGF-C/VEGFR3 pathway is protective in experimental colitis model. D'Alessio et al.'s [37] study shed light on the contribution of lymphatics to the pathogenesis of gut inflammation and suggested that correction of defective lymphatic function with VEGF-C has potential as a therapeutic strategy for IBD. VEGF165(b) (VEGF164(b) in mouse) is an antiangiogenic form of VEGF-A. Inflammation and angio- and lymphangiogenesis were reduced by expression of rVEGF164(b) in TNBS UC model [93]. Investigations about the VEGF signaling pathway are still continuing.

Leukocyte recruitment, adhesion molecules, and ligands may be the most studied area for the treatment of IBD. Monoclonal antibodies* natalizumab* (against *α*_4_ integrins) and* vedolizumab* (against *α*_4_ *β*_7_ integrin) were found to be effective and safe for induction of remission in CD in a meta-analysis [94]. However, side effects of natalizumab may be frustrating, including headache, fatigue, infusion reactions, and progressive multifocal leukoencephalopathy [95].* Etrolizumab* is a new anti-integrin antibody, targeting *β*_7_ integrin subunit. Vermeire et al. [96] completed etrolizumab phase 2 study with promising results for UC.* Alicaforsen* is an antisense ICAM-1 inhibitor which is in phase 2. Its local form is tested especially for UC. A lot of molecules have been studied for leukocyte trafficking and adhesion in IBD, for example, ATN-161, AJM300, AMG181, and CCX282-B [95, 97].

TSP is a well-known antiangiogenic molecule. Punekar et al. [98] demonstrated that angiogenesis and inflammation were blocked by TSP-1 and its mimetic peptide* ABT-510* in the murine model of IBD. Recently, it was reported that type 1 repeat domains of TSP-1 were exerting antiangiogenic effects [99].

Intestinal matrix is understood newly. This opens new research areas and therapeutic options for IBD. A humanized anti-gelatinase B antibody has entered phase I clinical trial. For fibrosis, targeting collagen cross-linking enzymes (LOX family) is a new interesting issue [61]. Other natural antiangiogenic molecules studied for the therapy of IBD are Ang1 [40], TGF-*β* [50], bFGF [53], HGF [57], Cav-1 [72], and endoglin [11]. But use of these molecules needs more time and study.* SMAD7* is an inhibitor of TGF-*β* signaling. An oral SMAD7 antisense oligonucleotide was found to be effective in a phase 2 clinical trial [100].

Nowadays, different therapeutic approaches are increased in IBD, at least experimentally.* HIF prolyl-hydroxylase inhibitors* have been investigated in murine model of IBD [101]. DNA vaccination and RNA interference targeting VEGF-A by using bacteria were demonstrated to reduce angiogenesis [102]. Inoculation of mice with* Bacillus polyfermenticus* as a probiotic aided healing of colitis by increasing angiogenesis via production of IL-8 in mucosa [103]. Poly-*γ*-glutamic acid (*γ*-PGA) is naturally secreted from various strains of* Bacillus* during the process of soybean fermentation. *γ*-PGA attenuated DSS-induced expression of VEGF-A and its receptor, VEGFR2. In addition, *γ*-PGA treatment led to reduced recruitment of leukocytes to the inflamed colon [104]. Also, probiotic yeast* Saccharomyces boulardii* can modulate angiogenesis to limit intestinal inflammation and promote mucosal tissue repair by regulating VEGFR signaling [105]. The enhanced endothelial CD146 expression promoted both angiogenesis and proinflammatory leukocyte extravasations. Using an anti-CD146 antibody, AA98, alone or together with an anti-TNF-*α* antibody significantly attenuated colitis and prevented colitis-associated colorectal carcinogenesis in mice [106]. Binion et al. [107] demonstrated that curcumin inhibited microvascular endothelial cell angiogenesis through inhibition of COX-2 expression and prostaglandin E2 production, suggesting that this natural product possessed antiangiogenic properties, which warrants further investigation as adjuvant treatment of IBD and cancer.

We do not know which factor initiates or mediates IBD. In the past, immune cells and their activation and relation were widely researched. The importance of nonimmune cells and intestinal microvasculature was understood, recently. Chronic intestinal inflammation is dependent on angiogenesis and this angiogenesis is modulated by immune system in IBD. In addition to immune-driven angiogenesis, activation of platelets, relationship of leukocyte and ECs, and hypercoagulation and dysfunction of endothelium are detected in intestinal microvasculature of patients with IBD.

## 7. Conclusion

Angiogenesis is a very complex process which includes multiple cell types, growth factors, cytokines, adhesion molecules, and signal transduction as mentioned above. They became new targets of IBD therapy. But while angiogenesis supports inflammation via leukocyte migration, carrying oxygen and nutrients, on the other hand, it has a major role in wound healing. As we summarized in this paper, probably all of the molecules have multiple effect and function as antiangiogenic and/or proangiogenic. The pathways of angiogenesis include its own inhibitor or modulator inside its cascade. Blocking one molecule generally leads to serious side effects due to blocking wound healing too. Angiogenic molecules look like perfect targets for the treatment of IBD, but they have risk for serious side effects because of their nature. The same molecule may behave diversely in different environments. There are a lot of stimulators, inhibitors, modulators, and mediators that effect only one event. This is a huge area for new research.
